# Individuals with high levels of autistic traits exhibit impaired cognitive but not affective theory of mind and empathy

**DOI:** 10.1002/pchj.727

**Published:** 2024-02-01

**Authors:** Bei‐lin Le, Yi‐hang Huang, Ling‐ling Wang, Hui‐xin Hu, Xuan Wang, Yi Wang, Ya Wang, Jia Huang, Simon S. Y. Lui, Raymond C. K. Chan

**Affiliations:** ^1^ Neuropsychology and Applied Cognitive Neuroscience Laboratory, CAS Key Laboratory of Mental Health, Institute of Psychology Chinese Academy of Sciences Beijing China; ^2^ Department of Psychology University of Chinese Academy of Sciences Beijing China; ^3^ Department of Psychiatry, School of Clinical Medicine The University of Hong Kong Hong Kong Special Administrative Region China

**Keywords:** autistic traits, empathy, theory of mind

## Abstract

Theory of mind (ToM) and empathy are considered key components of social cognition that are often impaired in individuals with autism spectrum disorders (ASD). However, it remains unclear whether individuals with high levels of autistic traits exhibit similar impairments in these two functions. This study examined the affective and cognitive domains of ToM and empathy in individuals with high levels of autistic traits. We recruited 84 participants with high levels and 78 participants with low levels of autistic traits to complete a set of self‐reported checklists and performance‐based tasks capturing affective and cognitive components of ToM and empathy. The results showed that participants with high levels of autistic traits exhibited significant impairments in cognitive but not in affective ToM and empathy compared with their counterparts with low levels of autistic traits. We also found that empathy impairments in people with high levels of autistic traits were confounded by alexithymia and depressive traits.

## INTRODUCTION

Autism spectrum disorders (ASD) are characterized by a wide range of impairments in social communication, restricted interests or behaviour, and repetitive, stereotyped actions (American Psychiatric Association, 2013). ASD typically emerge in early childhood, persist throughout life, and affect individuals' social and cognitive functions (Lai et al., 2014). Social cognition is a multifaceted construct, involving theory of mind (ToM) and empathy, among other aspects (Beaudoin & Beauchamp, 2020; Frith & Frith, 2007). ToM refers to the ability to understand the mental states and behaviour of oneself and others, while empathy concerns the ability to establish emotional and cognitive connections to resonate with others' experiences (Decety & Jackson, 2004; Premack & Woodruff, 1978).

Notably, several theories have been proposed to explain the social cognitive impairments in ASD, such as the Empathizing–Systemizing Theory and the Extreme Male Brain Theory, both suggesting that individuals with ASD possess different empathic abilities associated with an extreme masculinized brain (Baron‐Cohen et al., 1985). However, many prior studies were limited to isolated social cognitive aspects and viewed ToM and empathy as a unitary construct, without addressing this construct's affective and cognitive domains (Bird et al., 2010; Dziobek et al., 2008; Greenberg et al., 2023; Oakley et al., 2016).

In fact, both ToM and empathy comprise affective and cognitive domains (Davis, 1980; Shamay‐Tsoory et al., 2007), which can be captured using self‐reported or performance‐based measures (Devine & Hughes, 2013; Reniers et al., 2011; Stone et al., 1998). Cognitive ToM refers to the ability to make inferences about another person's beliefs and motivations, whilst affective ToM refers to the inferences one makes regarding others' emotions (Shamay‐Tsoory et al., 2007). Meanwhile, cognitive empathy involves speculating about another person's internal emotional and psychological states and understanding his/her emotions (Eisenberg et al., 1991), whereas affective empathy involves generating an emotional response to another person's situation and experiencing his/her emotions (Deutsch & Madle, 1975). The extant literature suggests that cognitive ToM and affective ToM are both significantly impaired in individuals with ASD (Zalla et al., 2009; Tin et al., 2018; Baldimtsi et al., 2020). For example, Tin et al. found that both Faux Pas inference of emotion (emotional ToM) and Faux Pas inference of intention (cognitive ToM) were particularly impaired in people with ASD (Tin et al., 2018). On the other hand, evidence suggests that cognitive but not affective empathy is impaired in individuals with ASD. For example, a previous study using the Questionnaire of Cognitive and Affective Empathy (QCAE; Reniers et al., 2011) showed a significant impairment in cognitive empathy and a less significant impairment in affective empathy in people with ASD (Shirayama et al., 2022). Other studies using the Interpersonal Reactivity Index (IRI; Davis, 1980) have also found that perspective‐taking (cognitive empathy) is significantly impaired, but emotional concern (affective empathy) remains intact in people with ASD (Mazza et al., 2014; Shirayama et al., 2022; McKenzie et al., 2022).

Autistic traits constitute a set of attenuated autistic symptoms found in the general population. Individuals with autistic traits resemble the typical behavioral and cognitive characteristics of people with ASD but fail to satisfy the diagnostic criteria for ASD (Constantino et al., 2003). Very little prior research has examined ToM and empathy in individuals with high levels of autistic traits. Recently, Shalev and Uzefovsky (2020) found that there was a correlation between empathic disequilibrium (ED) and autistic traits in 671 college students by measuring the ED with the empathy quotient (EQ) (Baron‐Cohen et al., 2003) and the IRI. Bohler et al. (2021) also found that the autism‐spectrum quotient (AQ) was negatively correlated with the EQ in 293 college students. Gillespie et al. (2017) measured ToM performance in 55 healthy adults by adopting the Movie for the Assessment of Social Cognition (MASC; Dziobek et al., 2006) and found that autism traits were negatively associated with ToM performance. However, these three studies (Bohler et al., 2021; Gillespie et al., 2017; Shalev & Uzefovsky, 2020) focused on either ToM or empathy alone and did not separately compare the ToM and empathy performance between people with high versus low levels of autistic traits. More importantly, few studies have been conducted to specifically examine the affective and cognitive domains of ToM and empathy in subclinical samples.

Notably, alexithymia and depressive traits might have an indirect effect on empathy (Butera et al., 2023; Jack & Murray, 2022; Yang et al., 2022). For example, Yang et al. (2021) recruited 1360 non‐clinical college students and adults and found alexithymia as a confound to empathic performances in individuals with autistic traits. Butera et al. (2023) found that the 35 youths with ASD and concomitant alexithymia whom they studied may experience emotional empathy differently from the 40 typically developing youth considered. On the other hand, Watanabe et al. (2021) reported that the degree of ASD traits was significantly associated with depressive traits in 151 medical college students, and their depressive traits were also correlated with their empathy deficits.

Taken together, previous studies suggest that individuals with ASD exhibit general impairments in empathy and ToM. However, few studies have examined the extent of empathy and ToM deficits using a cognitive–affective differential approach (Davis, 1980; Shamay‐Tsoory et al., 2007). Furthermore, many previous studies utilized a single measure to tap into social cognition in people with ASD, rather than using multiple measures (Gillespie et al., 2017; Shalev & Uzefovsky, 2020). More importantly, very few studies have specifically examined social cognitions in subclinical samples (Bohler et al., 2021; Gillespie et al., 2017; Shalev & Uzefovsky, 2020). To address these knowledge gaps, this study aimed to examine the affective and cognitive domains of ToM and empathy in individuals with high and low levels of autistic traits. We hypothesized that individuals with high levels of autistic traits would exhibit mild deficits in empathy and ToM. We also hypothesized that individuals with high levels of autistic traits would show impairment in cognitive rather than in affective ToM and empathy. Given the reported relationship of empathy with alexithymia and depressive symptoms, we further hypothesized that ToM and empathy impairments in people with high levels of autistic traits would disappear after controlling for the confounds of alexithymia and depressive symptoms.

## METHODS

### Participants

We recruited 4123 college students online and excluded those who self‐reported a family history of psychosis, history of neuropsychiatric conditions, or current neuropsychiatric conditions. The self‐reported AQ scale (Baron‐Cohen et al., 2001) was utilized to screen for individuals with high levels (score >30) and low levels (score ≤13) of autistic traits. Finally, we recruited 84 young adults with high levels of autistic traits (mean age = 21.73, SD = 3.65, range = 19–23 years) and 78 young adults with low levels of autistic traits (mean age = 21.29, SD = 2.38, range = 19–23 years) in this study.

The study was approved by the Ethics Committees of the Institute of Psychology, Chinese Academy of Sciences (protocol number: H20041). Informed consent was obtained from each participant. All the data were collected online owing to the college regulation policy during the COVID‐19 period. Each participant received 70 RMB ($10) upon completion of the study.

### Measures

The Yoni task is a computer‐based task (Shamay‐Tsoory et al., 2007) for measuring an individual's ability to judge others' mental states (ToM) based primarily on expression cues displayed on the face of the cartoon character Yoni. In our study, participants completed this task on their personal laptops, and data were collected online. We employed the Chinese version of the Yoni task (Ho et al., 2015), which has been used effectively in autism cohorts to assess ToM. This version assesses both first‐ and second‐order ToM abilities, dividing them into cognitive and affective ToM categories, as well as a physical control condition. This results in six distinct conditions: first‐order cognitive (Cog1), first‐order affective (Aff1), first‐order physical (Phy1), second‐order cognitive (Cog2), second‐order affective (Aff2), and second‐order physical (Phy2). First‐order ToM scenarios feature Yoni interacting with objects, while second‐order ToM scenarios incorporate an additional character. This task has been utilized in research involving individuals with autism (Shamay‐Tsoory et al., 2007) as it effectively measures both cognitive and affective ToM while remaining unaffected by any potential verbal limitations of participants with autism.

The Faux Pas task was adapted from the “Faux Pas recognition test” (Stone et al., 1998), which means “wrong act” and refers to a situation in which a speaker says something they should not have said, being unaware that it is inappropriate, and that may evoke unpleasant feelings. In our study, we invited participants to complete this task on their own laptops and we collected their data online. To assess higher‐order language‐based ToM, we used the Chinese version of the Faux Pas task (Zhu, Lee et al., 2007), which has high test–retest reliability (3 months, 0.83) and has been applied to both schizophrenia and autism patients. The details of this paradigm have been described elsewhere (Tin et al., 2018; Zhu, Lee et al., 2007). In short, the Faux Pas task generates four scores, which respectively reflect Faux Pas recognition, Faux Pas understanding, Faux Pas inference of emotion (affective ToM), and Faux Pas inference of intention (cognitive ToM). Finally, as a control for story comprehension, participants were asked a question about some important detail of the story (control question cut‐off score ≥9) (Zhu, Lee et al., 2007).

### Self‐report measures

The AQ is a 50‐item scale that assesses autistic traits across five domains: social skills, attention switching, attention to detail, communication, and imagination (Baron‐Cohen et al., 2001). The Chinese version of the AQ has demonstrated good psychometric properties and a Cronbach's alpha of 0.63 (Lau et al., 2013). We adopted the same categorization method and AQ cut‐off scores as Stevenson and Hart (2017) in this study; that is, those participants with an AQ score >30 were classified into the high autistic trait group, and those with an AQ score <13 were classified into the low autistic trait group.

The 31‐item QCAE evaluates empathy (Reniers et al., 2011), and its Chinese version shows reliable internal consistency (*α* = 0.86) (Liang et al., 2019). The QCAE measures two factors, namely cognitive empathy and affective empathy, with higher scores indicating better empathic ability.

The IRI was utilized to further validate empathic abilities (Davis, 1980). The Chinese translation of the IRI has shown robust construct validity and internal consistency reliability (*α* = 0.492–0.758; Zhang et al., 2010). The IRI comprises two factors: perspective‐taking (PT) and empathic concern (EC). The PT factor evaluates the cognitive aspect, while the EC factor measures the affective component.

The Patient Health Questionnaire‐9 (PHQ‐9) was employed to assess depressive traits (Kroenke et al., 2001). The Chinese translation of the PHQ‐9 displays strong construct validity and internal consistency reliability (*α* = 0.89–0.91; Chen et al., 2010).

The 20‐item Toronto Alexithymia Scale (TAS) measures alexithymia (Bagby et al., 1994) and includes three subscales: difficulty in identifying feelings, difficulty in describing feelings, and externally oriented thinking. The Chinese version was adopted (Zhu, Yi et al., 2007), and the current dataset's Cronbach's alpha for the TAS is 0.84.

### Statistical analyses

Demographic information and scores of measures and questionnaires were analyzed using the Statistical Package for Social Science (SPSS; version 22.0) (IBM Corp., 2013). The level of significance was set at *p <* .05 (two‐tailed), unless otherwise specified. Independent sample *t* tests were conducted between the high and low autistic subgroups to detect group differences across all demographic information. To control for the potential influence of depression and alexithymia traits on ToM and empathy performance, group differences in ToM and empathy performance were examined using multiple univariate analysis with the PHQ or TAS scale score set as a covariate.

## RESULTS

### Overall performances

Our final sample comprised 84 participants with high levels and 78 participants with low levels of autistic traits. However, several recruited participants failed to complete the Yoni task (*n* = 6) or the Faux Pas task (*n* = 15) correctly (i.e., scored lower than 9 in the Faux Pas control questions) (Zhu, Lee et al., 2007), resulting in missing data in our sample.

The groups with high and low levels of autistic traits did not differ in sex (*p* = .117), age (*p* = .379), and education (*p* = .617) (see Table 1). As expected, the two groups differed significantly in the AQ (*p* < .001), PHQ (*p* < .001), and TAS (*p* < .001) scores (see Table 1).

**TABLE 1:** Demographics profile of the participants.

Variables	High autistic traits group (*n* = 84)		Low autistic traits group (*n* = 78)		*T*/*χ* ^*2*^	*df*	*p‐*value
	Mean	*SD*	Mean	*SD*			
Sex (male vs. female)	37 vs. 47		25 vs. 53		2.46	1	.117
Age (years)	21.73	3.65	21.29	2.38	0.83	160	.379
Length of education (years)	14.82	2.11	14.65	1.99	0.50	160	.617
AQ	29.86	5.20	13.96	4.93	19.93	160	**<.001**
PHQ	9.48	5.57	4.56	4.25	6.33	160	**<.001**
TAS	60.75	9.35	45.49	11.55	9.27	160	**<.001**

*Note*: The sex ratio comparison used the *χ* ^2^ test. Statistically significant differences are in bold face.

Abbreviations: AQ = autism‐spectrum quotient; PHQ = Patient Health Questionnaire; TAS = Toronto Alexithymia Scale.

### Comparison of empathy between groups with high and low levels of autistic traits

The high autistic trait group exhibited significantly lower scores in cognitive empathy on the QCAE (*p* < .001) than the low autistic trait group (see Table 2). However, the two groups showed comparable QCAE affective empathy scores (*p* = .461) (see Table 2). The high autistic trait group exhibited significantly lower scores in IRI perspective taking (cognitive empathy) (*p* = .003) (see Table 2) than the low autistic trait group, but with no significant difference in IRI emotional concern (affective empathy) (*p* = .739) (see Table 2). However, the significant group difference in IRI perspective taking (cognitive empathy) disappeared after controlling for PHQ score (*p* = .093) (see Table 3) or TAS score (*p* = .699), respectively (see Table 4).

**TABLE 2:** Comparison tests of empathy and ToM between groups with high and low levels of autistic traits (without controlling covariates).

	High autistic traits group (*n* = 84)		Low autistic traits group (*n* = 78)		*F*‐value	*df*	*p*‐value	Partial eta‐squared
	Mean	*SD*	Mean	*SD*				
QCAE_Cognitive empathy	52.70	0.75	59.82	0.78	43.27	(1160)	**<.001**	0.213
QCAE_Affective empathy	34.27	0.55	34.86	0.57	0.55	(1160)	.461	0.003
IRI_Perspective taking	13.31	0.33	14.76	0.34	9.21	(1160)	**.003**	0.054
IRI_Personal distress	13.02	0.41	10.42	0.42	19.78	(1160)	**<.001**	0.110
IRI_Fantasy	15.26	0.39	17.10	0.41	10.63	(1160)	**.001**	0.062
IRI_Empathic concern	15.93	0.39	16.12	0.40	0.11	(1160)	.739	0.001
Yoni task	(*n* = 80)		(*n* = 76)					
Yoni_Cog1	0.91	0.02	0.91	0.02	<0.01	(1154)	.948	<0.001
Yoni_Aff1	0.92	0.02	0.94	0.02	0.46	(1154)	.499	0.003
Yoni_Phy1	0.86	0.03	0.90	0.03	1.20	(1154)	.275	0.008
Yoni_Cog2	0.83	0.02	0.85	0.02	0.93	(1154)	.336	0.006
Yoni_Aff2	0.83	0.01	0.86	0.02	1.76	(1154)	.187	0.011
Yoni_Phy2	0.91	0.02	0.95	0.02	2.36	(1154)	.127	0.015
Faux Pas task	(*n* = 76)		(*n* = 71)					
FP recognition	8.37	0.18	8.41	0.19	0.02	(1145)	.878	<0.001
FP understanding	7.79	0.22	7.79	0.23	<0.01	(1145)	.998	<0.001
FP inference of intention	2.66	0.32	4.27	0.33	12.39	(1145)	**.001**	0.079
FP inference of emotion	5.33	0.26	6.32	0.27	7.04	(1145)	.009	0.046
FP control	9.86	0.05	9.85	0.05	0.02	(1145)	.881	<0.001

*Note*: Statistically significant differences are in bold face (after Bonferroni correction).

Abbreviations: FP = Faux Pas task; IRI = interpersonal reactivity index; QCAE = Questionnaire of Cognitive and Affective Empathy; Yoni_Aff1 = first degree affective theory of mind; Yoni_Aff2 = second degree affective theory of mind; Yoni_Cog1 = first degree cognitive theory of mind; Yoni_Cog2 = second degree cognitive theory of mind; Yoni_Phy1 = first degree physical control; Yoni_Phy2 = second degree physical control.

**TABLE 3:** Comparison tests of empathy and ToM between groups with high and low level of autistic traits (controlling for PHQ as a covariate).

	High autistic traits group (*n* = 84)		Low autistic traits group (*n* = 78)		*F*‐value	*df*	*p*‐value	Partial eta‐squared
	Mean	*SD*	Mean	*SD*				
QCAE_Cognitive empathy	53.34^a^	0.78	59.13^a^	0.81	23.72	(1159)	**<.001**	0.130
QCAE_Affective empathy	34.20^a^	0.58	34.94^a^	0.61	0.70	(1159)	.406	0.004
IRI_Perspective taking	13.58^a^	0.35	14.47^a^	0.36	2.86	(1159)	.093	0.018
IRI_Personal distress	12.67^a^	0.42	10.81^a^	0.44	8.43	(1159)	.004	0.050
IRI_Fantasy	15.48^a^	0.41	16.87^a^	0.43	4.92	(1159)	.028	0.030
IRI_Empathic concern	16.18^a^	0.41	15.85^a^	0.43	0.29	(1159)	.593	0.002
Yoni task	(*n* = 80)		(*n* = 76)					
Yoni_Cog1	0.92^a^	0.02	0.90^a^	0.02	0.63	(1153)	.429	0.004
Yoni_Aff1	0.94^a^	0.02	0.92^a^	0.02	0.22	(1153)	.639	0.001
Yoni_Cog2	0.84^a^	0.02	0.83^a^	0.02	0.14	(1153)	.711	0.001
Yoni_Aff2	0.84^a^	0.02	0.85^a^	0.02	0.03	(1153)	.857	<0.001
Faux Pas task	(*n* = 76)		(*n* = 71)					
FP recognition	8.28^a^	0.19	8.50^a^	0.20	0.56	(1144)	.455	0.004
FP understanding	7.80^a^	0.23	7.77^a^	0.24	0.01	(1144)	.930	<0.001
FP inference of intention	2.51^a^	0.34	4.42^a^	0.35	13.76	(1144)	**<.001**	0.087
FP inference of emotion	5.37^a^	0.28	6.28^a^	0.29	4.61	(1144)	.033	0.031

*Note*: Statistically significant differences are in bold face (after Bonferroni correction).

Abbreviations: FP = Faux Pas task; IRI = interpersonal reactivity index; QCAE = Questionnaire of Cognitive and Affective Empathy; Yoni_Aff1 = first degree affective theory of mind; Yoni_Aff2 = second degree affective theory of mind; Yoni_Cog1 = first degree cognitive theory of mind; Yoni_Cog2 = second degree cognitive theory of mind.

^a^ Represents the estimated mean values after adjustment based on the covariant PHQ (the Patient Health Questionnaire).

**TABLE 4:** Comparison tests of empathy and ToM between groups with high and low levels of autistic traits (controlling for TAS as a covariate).

	High autistic traits group (*n* = 84)		Low autistic traits group (*n* = 78)		*F*‐value	*df*	*p*‐value	Partial eta‐squared
	Mean	*SD*	Mean	*SD*				
QCAE_Cognitive empathy	54.21^a^	0.80	58.20^a^	0.84	9.71	(1159)	**.002**	0.058
QCAE_Affective empathy	34.10^a^	0.62	35.05^a^	0.65	0.94	(1159)	.335	0.006
IRI_Perspective taking	13.90^a^	0.36	14.12^a^	0.37	0.15	(1159)	.699	0.001
IRI_Personal distress	11.83^a^	0.41	11.71^a^	0.43	0.03	(1159)	.853	<0.001
IRI_Fantasy	15.54^a^	0.44	16.81^a^	0.46	3.30	(1159)	.071	0.020
IRI_Empathic concern	16.41^a^	0.43	15.60^a^	0.45	1.41	(1159)	.238	0.009
Yoni task	(*n* = 80)		(*n* = 76)					
Yoni_Cog1	0.90^a^	0.02	0.93^a^	0.02	0.51	(1153)	.475	0.003
Yoni_Aff1	0.92^a^	0.02	0.94^a^	0.02	0.45	(1153)	.504	0.003
Yoni_Cog2	0.84^a^	0.02	0.84^a^	0.02	0.05	(1153)	.822	<0.001
Yoni_Aff2	0.83^a^	0.02	0.86^a^	0.02	0.82	(1153)	.367	0.005
Faux Pas task	(*n* = 76)		(*n* = 71)					
FP recognition	8.26^a^	0.21	8.52^a^	0.21	0.61	(1144)	.438	0.004
FP understanding	7.76^a^	0.25	7.82^a^	0.26	0.02	(1144)	.891	<0.001
FP inference of intention	2.38^a^	0.36	4.57^a^	0.38	14.32	(1144)	**<.001**	0.090
FP inference of emotion	5.25^a^	0.30	6.41^a^	0.31	5.82	(1144)	.017	0.039

*Note*: Statistically significant differences are in bold face (after Bonferroni correction).

Abbreviations: FP = Faux Pas task; IRI = interpersonal reactivity index; QCAE = Questionnaire of Cognitive and Affective Empathy; Yoni_Aff1 = first degree affective theory of mind; Yoni_Aff2 = second degree affective theory of mind; Yoni_Cog1 = first degree cognitive theory of mind; Yoni_Cog2 = second degree cognitive theory of mind.

^a^ Represents the estimated mean values after adjustment based on the covariant TAS (the Toronto Alexithymia Scale).

### Comparison of ToM between groups with high and low levels of autistic traits

As shown in Table 2, the two groups did not differ in the Yoni task control (physical) conditions (Phy1: *p* = .256; Phy2: *p* = .141) (see Table 2), suggesting that participants were able to understand and follow the task instructions. Furthermore, the two groups did not differ in first‐level cognitive ToM (*p* = .948), first‐level affective ToM (*p* = .499), second‐level cognitive ToM (*p* = .336), or second‐level affective ToM (*p* = .187) in the Yoni task (see Table 2).

Regarding the Faux Pas task, we found that the two groups performed similarly well in the control condition (*p* = .881), indicating that participants could understand and follow the task instructions. The group difference in the Faux Pas inference of intention (cognitive ToM) (*p* = .001) reached the Bonferroni‐adjusted significance threshold (*p* = .05/17), but it did not reach in the Faux Pas inference of emotion (affective ToM) (*p* = .009) (see Table 2). In addition, the group differences in Faux Pas recognition (*p* = .878) and Faux Pas understanding (*p* = .998) both failed to reach the Bonferroni‐adjusted significance threshold (see Table 2).

Furthermore, the significant group difference in the Faux Pas inference of intention (cognitive ToM) did not appear to be confounded by depressive traits (with PHQ as a covariate) (*p* < .001) (see Table 3) and alexithymia (with TAS as a covariate) (*p* < .001) (see Table 4).

## DISCUSSION

Our findings suggest that people with high levels of autistic traits have mild impairments in ToM and empathy, affecting the cognitive rather than the affective aspects. Moreover, cognitive empathy impairment in people with high levels of autistic traits was confounded by alexithymia and depressive traits, consistent with our hypotheses.

Our findings of deficits in cognitive empathy in the high autistic trait group are consistent with prior evidence. Prior research using the QCAE has shown significant impairments in cognitive empathy in individuals with ASD, while affective empathy is impaired to a lesser extent (Bird & Cook, 2013). Likewise, our findings of impaired perspective‐taking (cognitive empathy) in the high autistic trait group are consistent with previous results (Mazza et al., 2014). Recently, some studies have reported that alexithymia and depression traits could indirectly affect one's ToM and empathy (Jack & Murray, 2022). This notion is supported by our findings. Specifically, we found that the significant group difference in cognitive empathy disappeared after taking into account the confounding effects of depressive traits and alexithymia. Such a finding agrees with the results of a previous study (Santiesteban et al., 2021), which showed that alexithymia contributed mostly to the empathy‐related deficits in ASD. Future research on empathy should further investigate the mechanisms by which alexithymia and depressive traits could influence cognitive and affective empathy. Taken together, our results suggest that the high autistic trait group has poorer cognitive but not affective empathy, similar to the case for clinical patients with ASD (Baron‐Cohen & Wheelwright, 2004; Oakley et al., 2016), and that depressive traits and alexithymia are possible reasons for such empathy deficits in the ASD spectrum.

Regarding ToM, our findings of cognitive rather than affective ToM impairments in the high autistic trait group also concur with the literature on clinical ASD samples. Previous studies showed significant impairments in cognitive ToM in ASD patients (Baron‐Cohen et al., 1985; Happé, 1994). For example, Tin et al.'s study found significant impairments in all four subcategories of the Faux Pas test in ASD patients, with particularly large impairments in the inference of intention (cognitive ToM) subcategories (Tin et al., 2018). Our findings appear to show similar patterns of Faux Pas performance in the high autistic trait group. However, we did not find any significant group differences in scores for Faux Pas intention of emotion, Faux Pas recognition, and Faux Pas understanding between the two groups of high and low levels of autistic traits. The milder severity of ToM deficits in subclinical samples than in clinical samples may explain this expected finding. In relation to the Yoni task, our findings do not suggest that people with high autistic traits have any impairment in first‐ or second‐level ToM, or in cognitive ToM or affective ToM. Such negative findings may be attributable to the low task difficulty of the paradigm. A previous study (Tin et al., 2018) employed mixed model analyses of variance (ANOVAs) to study the difficulty of the Yoni and Faux Pas tasks and reported that the Faux Pas inference of emotion and inference of intention both had higher task difficulty than the Yoni task, which could possibly have contributed to our negative findings. Taking the Faux Pas and Yoni task results together, people with high autistic traits appear to have poorer cognitive but not affective ToM.

Our findings may have clinical implications. Future research could study whether training on cognitive ToM in people with high levels of autistic features could improve social functioning and reduce the risk of the development of other psychiatric disorders such as depression in this subclinical population. Moreover, given that alexithymia and depressive symptoms influence cognitive ToM in people with high levels of autistic features, these psychopathologies should be considered when developing cognitive training for ToM.

However, several limitations should be considered. First, other social and nonsocial cognitive abilities (such as facial emotion perception ability and executive function) could influence empathy and ToM (Livingston & Happé, 2017; Oakley et al., 2016) but were not included in our study. Second, all the data were collected online during the COVID period, because the public health policies at the time precluded face‐to‐face data collection. Finally, the reliability estimate of the AQ in our sample was only modest. According to Baron‐Cohen et al. (2006), future studies comparing levels of autistic traits should utilize both the parent‐ and the self‐reported versions of AQ.

To conclude, people with high levels of autistic traits exhibited poorer cognitive but not poorer affective empathy and ToM than people with low levels of autistic traits. Such cognitive empathy deficits were confounded by alexithymia and depressive traits. Future research should explore the underlying mechanisms of these deficits and investigate the correlation between other social or nonsocial deficits with cognitive and affective ToM and empathy.

## CONFLICT OF INTEREST STATEMENT

The authors have no conflicts of interest to declare that are relevant to the content of this article.

## ETHICS STATEMENT

This study was approved by the Ethics Committee of the Institute of Psychology of the Chinese Academy of Sciences (H20041). All participants gave written informed consent.
